# Cutaneous Manifestations of Langerhans Cell Histiocytosis in Pediatric Age: A Case Report

**DOI:** 10.7759/cureus.70141

**Published:** 2024-09-25

**Authors:** Catarina Afonso, Tiago Dias, Carlos Teixeira, David Rodrigues, Marília Mira

**Affiliations:** 1 General Practice/Family Medicine, Unidade de Saúde Familiar Planície, Unidade Local de Saúde do Alentejo Central, Évora, PRT; 2 Nursing, Escola Superior de Enfermagem São João de Deus, Universidade de Évora, Évora, PRT; 3 General Practice/Family Medicine, Unidade de Saúde Familiar Alcaides, Unidade Local de Saúde do Alentejo Central, Montemor o Novo, PRT; 4 Nursing, Unidade de Saúde Familiar Planície, Unidade Local de Saúde do Alentejo Central, Évora, PRT

**Keywords:** child, clinical case report, family medicine/general practice, langerhans cell histiocytosis (lch), pediatric dermatology, skin lesions

## Abstract

Langerhans cell histiocytosis (LCH) is a rare histiocytic neoplastic disorder that mainly affects the skin and bones, with dermatological manifestations that can be easily confused with other dermatological conditions, such as seborrheic eczema, psoriasis, lesions of herpes simplex virus infection, fungal infection, lichen planus, and cutaneous lymphomas. This case report describes an eight-month-old infant who, at a child health appointment, presented with progressive erythematous papulovesicular lesions, initially treated with hygiene measures (skin hydration and hygiene) and mupirocin ointment, but which persisted and worsened, leading to a skin biopsy. The diagnosis of self-limiting congenital histiocytosis was confirmed, and the child was referred to the Portuguese Oncology Institute. The case highlights the importance of early recognition of LCH, the multidisciplinary approach, and the crucial role of the family doctor in the diagnosis and appropriate management of this rare condition.

## Introduction

Langerhans cell histiocytosis (LCH) is a histiocytic neoplastic disorder that affects essentially skin and bones, but might as well affect other organs [1]. Although its etiology remains unknown, genetics and environmental triggers seem to play a role together. The incidence of LCH is estimated to be one to two cases per million per year in adults and three to five per million per year in children [2,3]. It can appear at any age, but peak incidence can be found between one- and three-year-old children, and it is more common in white males [2,4]. Being a rare and heterogeneous disease, current knowledge about its etiology, clinical evolution, and treatment is scarce [3].

A delay in diagnosis can happen frequently due to a very variable clinical presentation. LCH can limit manifestations to a single organ, but can also have a multisystemic involvement [5]. The acute disseminated multisystemic form is more frequent in children under three years of age, while the single organ form is more common among older children and adults [5]. The most affected areas in children are bone, skin, and lymphatic ganglia, by decrescent order [5].

Skin involvement occurs in approximately 40% of patients, with the most common cutaneous manifestations being an eczematous eruption resembling a Candida infection, as well as brown or purplish papules [6]. Other cutaneous manifestations that can be found are pustular, petechial, vesicular, and papule-nodular lesions [6]. Several dermatological conditions may resemble the skin lesions of LCH, making diagnosis challenging. These include seborrheic dermatitis, psoriasis, atopic dermatitis, and viral rashes, which can mimic the eczematous or papular nature of the lesions.

The diagnosis of LCH is established by correlating clinical manifestations with anatomopathological and immunohistochemistry findings, with a particular emphasis on biopsy results [1]. The most common manifestations include bone lesions and rashes. However, specific signs and symptoms may appear in some organs.

General practice plays a crucial role in monitoring children and detecting diseases in their early stages, as it monitors patients in a regular and holistic manner. This clinical case description highlights the wide diversity of symptoms correlated with this condition, as it emphasizes the inherent complexity in the diagnostic process and in providing healthcare to the family. The main goals of this case are to increase knowledge among the healthcare community about this rare condition; emphasize the importance of remaining suspicious regarding signs and symptoms that don’t match with already established diagnosis and highlight the importance the general practitioner has in the early assessment of patients.

This article was previously announced as an oral communication at the 41st General Practice Medicine National Encounter of the General Practice Portuguese Association, which occurred between April 3rd and April 6th, 2024.

## Case presentation

Female patient, eight months old, belonging to a nuclear family in Duvall stage V, living with her parents and two brothers (aged three and 17), with a personal history of dystocic delivery (cesarean section) at 38 weeks and subsequent physiological jaundice with spontaneous remission at her 14th day of life and without chronic diseases, known allergies, previous hospitalizations, and no significant family history. She takes oral supplementation with 400 international units (IU) per day of vitamin D. Since then, she has been monitored regularly by her general practitioner and has had adequate height, weight, and psychomotor development. She has accomplished the Portuguese National Vaccination Program (PNV) and received an extra-PNV rotavirus vaccine at two months of age.

When she was six weeks old, she presented to her general practitioner with erythematous papulovesicular lesions, apparently not pruritic, that first appeared on her head seven days before and then progressed downwards. She initially formed vesicular lesions that evolved later to papules and crusts by the latter, so prurigo strophulus was considered. In the inguinal fold area, there were more concentrated, superficial pustule lesions and inflammatory nodules, more compatible with a folliculitis hypothesis. The parents denied any other signs or symptoms, including fever or upper respiratory tract infections, and without relevant epidemiological context. Cutaneous hydration and hygiene general measures were promoted, as well as an empirical prescription of a mupirocin ointment, twice daily, for seven days.

**Figure 1:**
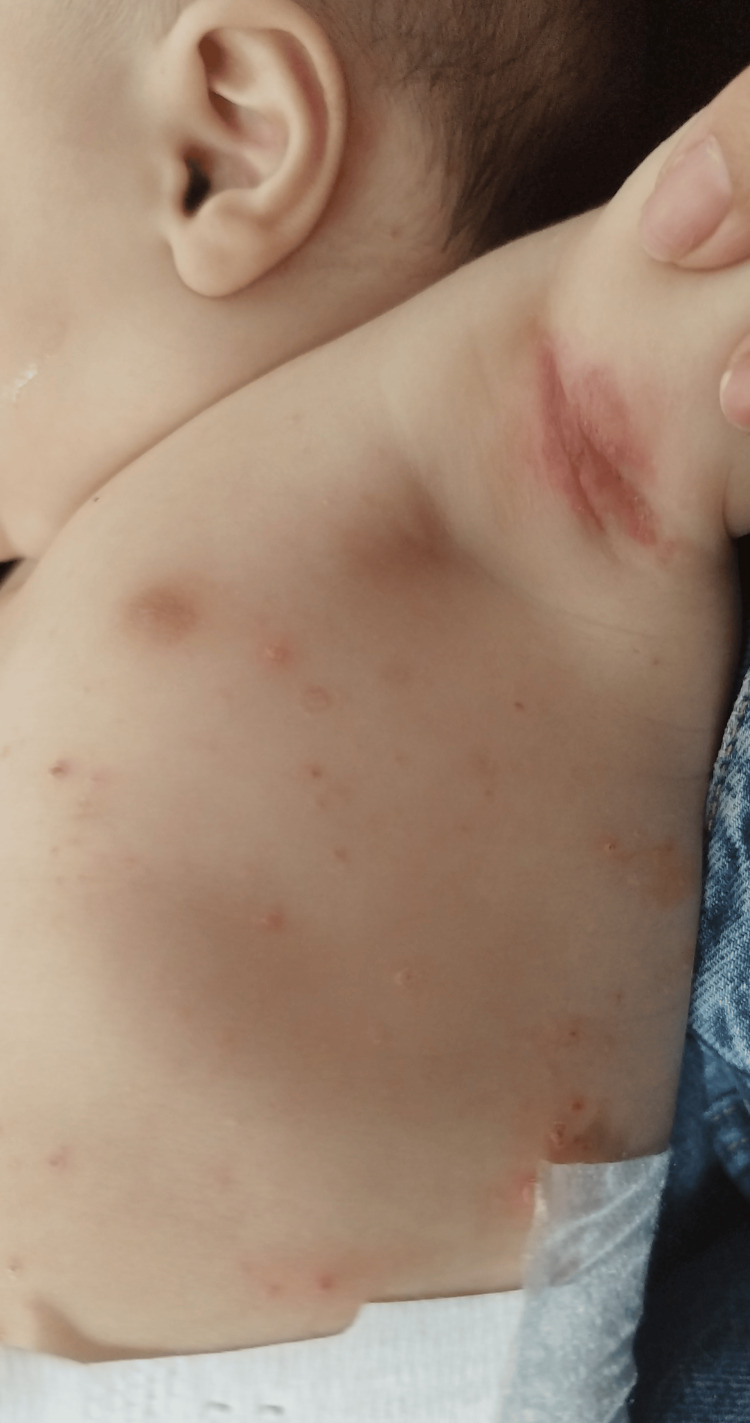
Erythematous papulovesicular lesions scattered in the trunk and more converged in the axillary region.

She came back to her two-month-old appointment, and she maintained the papulovesicular lesions and herpetic clusters in the inguinal region bilaterally, with scarring and spontaneous remission of the lesions, sparing the palms, and soles (Figure 1). She also presented with bilateral axillary and inguinal palpable adenopathies, characterized by small, mobile, and soft nodes, without tenderness. No abdominal organomegaly was noted. She was then referred to a dermatology appointment at her resident hospital. A revaluation appointment was also scheduled and symptomatic treatment with moisturizing, repairing, and calming cream was prescribed.

**Figure 2:**
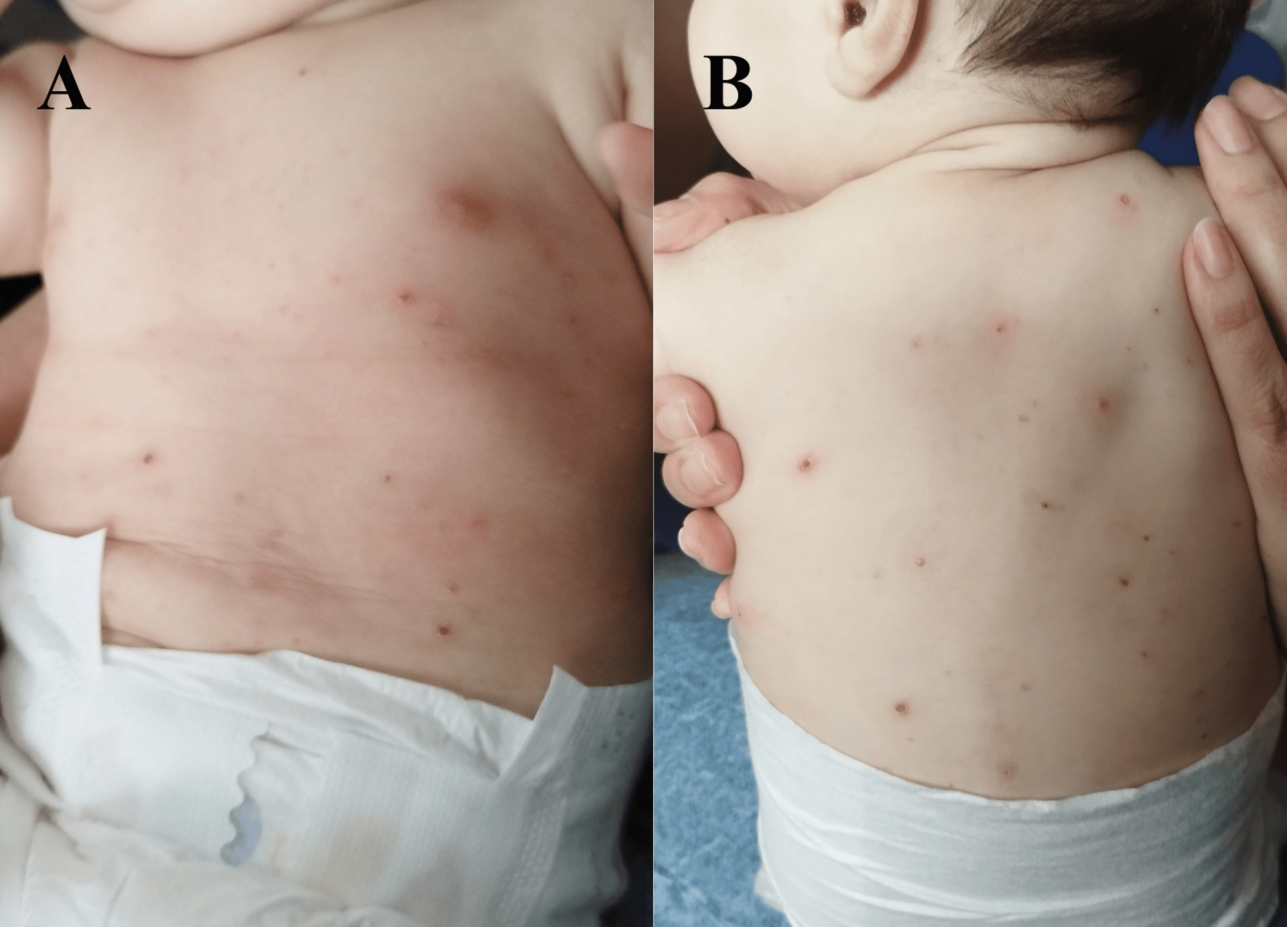
Skin lesions scattered across the trunk and back. (A) Papulovesicular eruptions with an erythematous base and crusts located on the trunk region; (B) Papulovesicular eruptions with an erythematous base and crusts located on the back.

She was the same when she came back (Figure 2). Since the clinical condition wasn’t getting any better and the dermatology appointment was taking too much time to be scheduled, the parents decided to address a dermatology appointment at a private healthcare center. She then performed one and two herpes simplex virus (HSV) PCR, bacteriological, and mycological analysis, which were all negative, excluding herpetic eczema and candidiasis. In spite of those results, it was prescribed fluconazole 40 mg/mL, 0.4 mL once daily for 14 days, and a mupirocin ointment, twice daily, for 14 days, with no subsequent improvements.

**Figure 3:**
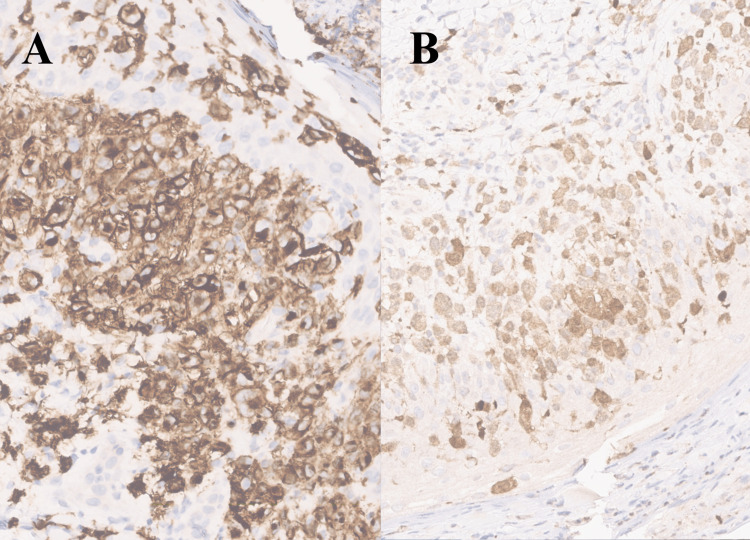
Immunohistochemistry of abdominal dermis biopsy with positive CD1a (A) and S100 (B) staining.

A cutaneous biopsy was performed and revealed a “moderately dense infiltrate, in the superficial dermis, with epidermotropism, consisting of histiocytes (...) with positive CD1a and S100 staining and negative CD68, aspects compatible with a diagnosis of self-limited congenital histiocytosis” (Figure 3). Hence, a cutaneous biopsy confirmed the LCH hypothesis, and samples were sent to a specialized hospital service in order blades could be reviewed. It was prescribed topic corticotherapy (mometasone furoate 1 mg/g cream, once daily for 14 days) and a repairing, hydrating cream.

The patient is currently being monitored regularly at the Oncology Portuguese Institute, receiving a multidisciplinary approach that includes evaluations by hematology, pediatric oncology, and pediatrics. She has done blood tests routinely, where a high alkaline phosphatase of 1127 U/L and a G immunoglobulin of 1071 U/L stand out. At present, a conservative approach is recommended.

The patient has also kept regular appointments with her general practitioner over time, with the frequency recommended by the Portuguese National Child and Youth Health Program. The parents were initially cautious about PNV vaccination since they associated a worsening of the rash with a rotavirus vaccine dose that was taken two days earlier. The hospital attendant physician and the general practitioner, however, clarified the parents and hence they let the patient take all the vaccines provided by the PNV, and there were no intercurrences to be registered.

A familial evaluation was performed and it centered on clinical history, family structure, family life cycle’s phase, transgenerational repetitions of patterns, significant life events, relationship patterns, and familial balance. A genogram was executed and interpreted in order to help with the evaluation. Besides, individual healthcare appointments for both parents and both brothers were conducted. The primary goal of those appointments was to provide support for change and hence focus on the prevention of mental health illness. In the comprehensive assessment of the disease, environmental exposures were also considered and subsequently excluded as potential contributing factors.

During the patient’s follow-up in the different stages of her illness, her parents were offered the possibility of psychological support, as there were periods of increased stress, anxiety, and sadness. They have so far recused specialized psychological support, but they maintain regular contact with their general practitioner.

The patient continues her scheduled follow-up, while the family doctor remains vigilant for any new signs and symptoms, as well as attentive to family dynamics.

**Figure 4:**
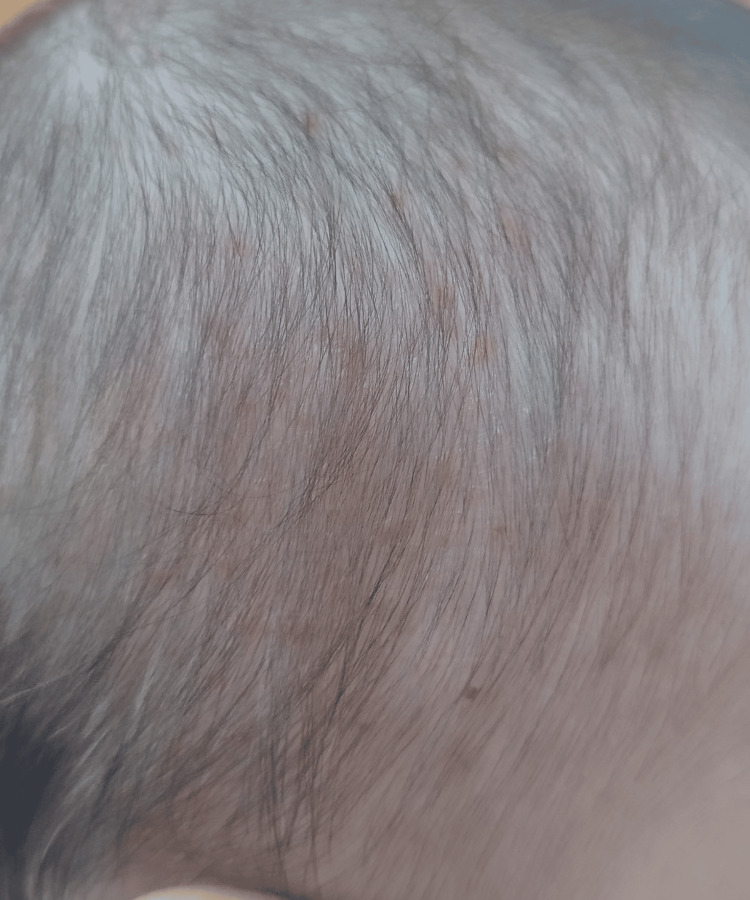
Crust forms non-active lesions on the scalp.

At the moment, the patient is stable, without active lesions (Figure 4). The mother reported that occasionally uses topical corticosteroid (mometasone furoate cream) once daily in the lesions, for a maximum duration of one week, with remission of symptoms. She began to diversify her diet without complications and is progressing satisfactorily in terms of height and weight, with neurodevelopment and behavior appropriate for her age.

## Discussion

LCH is a condition that affects majorly children and young adults and it can affect any organ and it can affect several simultaneously, like bones, lungs, skin, oral and genital mucosae, and endocrine glandules [7]. Being a rare, very heterogenous disease with a wide range of symptoms, early diagnosis is usually difficult.

Prognosis is intimately connected to the age of onset and to the extent of involving organs. Age of onset inferior to two years and systemic involvement are the bad prognostic predictors [8]. One has to be alert and sensibilized since it is rare.

Differential diagnosis can be a great challenge. Dermatological manifestations can be misunderstood with seborrheic eczema, psoriasis, herpes simplex virus infection lesions, lichen planus, and cutaneous lymphomas [9]. Besides, lymphadenopathy is present in up to 20% of patients, being more common in children than in adults [5,10].

As for what concerns vaccination, the connection between LCH and vaccines is not well established, since there are studies that don’t recommend the administration of live vaccines, while others don’t counter indicate. The evaluation of an experienced healthcare professional is important to consider the risk-benefit relation of its administration.

It is possible to identify, thanks to this clinical case, the obstacles to a correct and attempted diagnosis of LCH, since the wide range of dermatological manifestations can be easily misunderstood with other pathologies.

Diagnosis is based on LHC pathological evidence in the context of suggestive clinical and imaging features [1]. In this clinical case, it was possible to follow the various challenges of clinical suspicion, revealing the need to perform skin biopsies in order to assist in medical diagnosis. The treatment depends on the extension, gravity, and organs involved. When there’s only skin involvement, spontaneous resolution is common [11]. The treatment can include a simple conservative approach, observing, monitoring, and performing regular analysis, as it happened with this case, or it can involve systemic treatments, that are reserved to the multisystemic form.

The general practitioner plays a crucial role in guiding the case, emphasizing the importance of a complete evaluation that takes into account familial and social aspects. A holistic approach to the patient family context contributes to a better management of the condition. Family is a source of support during the acute phase and on the adaptation to the chronic phase, especially when it concerns children. Hence, priority was given to a family assessment and continued monitoring of both the user and the family.

## Conclusions

In summary, we report a rare case of LCH intriguing dermatologic manifestations in nursling. This case highlights the importance of a careful and comprehensive approach to the diagnosis of rare diseases. Atypical skin lesions challenge diagnosis, requiring a multidisciplinary approach for proper disease management. The complexity of LCH diagnosis, especially in cases with restricted manifestations to the skin, emphasizes the need to improve medical knowledge about rare conditions.

This case also reinforces the crucial role of the general practitioner in the initial assessment and continuity of care, ensuring that patients receive comprehensive and integrated care.

## References

[1] Utiyama TO, Malzoni ML, Vasques TG, Gomes CT (2023). Langerhans cell histiocytosis: A rare case of the multisystemic form in an infant. An Bras Dermatol.

[2] Goyal G, Shah MV, Hook CC, Wolanskyj AP, Call TG, Rech KL, Go RS (2018). Adult disseminated Langerhans cell histiocytosis: Incidence, racial disparities and long-term outcomes. Br J Haematol.

[3] Brito MG, Martins A, Andrade J, Guimarães J, Mariz J (2014). Adulthood Langerhans cell histiocytosis: Experience of two Portuguese hospitals. Acta Med Port.

[4] Nicholson HS, Egeler RM, Nesbit ME (1998). The epidemiology of Langerhans cell histiocytosis. Hematol Oncol Clin North Am.

[5] Grois N, Pötschger U, Prosch H (2006). Risk factors for diabetes insipidus in langerhans cell histiocytosis. Pediatr Blood Cancer.

[6] Newman B, Hu W, Nigro K, Gilliam AC (2007). Aggressive histiocytic disorders that can involve the skin. J Am Acad Dermatol.

[7] Subramaniyan R, Ramachandran R, Rajangam G, Donaparthi N (2015). Purely cutaneous Langerhans cell histiocytosis presenting as an ulcer on the chin in an elderly man successfully treated with thalidomide. Indian Dermatol Online J.

[8] Gadner H, Grois N, Arico M (2001). A randomized trial of treatment for multisystem Langerhans' cell histiocytosis. J Pediatr.

[9] Girschikofsky M, Arico M, Castillo D (2013). Management of adult patients with Langerhans cell histiocytosis: Recommendations from an expert panel on behalf of Euro-Histio-Net. Orphanet J Rare Dis.

[10] Ravindran A, Goyal G, Failing JJ, Go RS, Rech KL (2018). Florid dermatopathic lymphadenopathy—A morphological mimic of Langerhans cell histiocytosis. Clin Case Rep.

[11] Krooks J, Minkov M, Weatherall AG (2018). Langerhans cell histiocytosis in children: Diagnosis, differential diagnosis, treatment, sequelae, and standardized follow-up. J Am Acad Dermatol.

